# Knowledge, perception, attitude, and practice of complementary and alternative medicine by health care workers in Garki hospital Abuja, Nigeria

**DOI:** 10.1186/s12906-024-04429-x

**Published:** 2024-05-09

**Authors:** Enole Jennifer Onche, Mojisola Morenike Oluwasanu, Yetunde Olufisayo John-Akinola

**Affiliations:** Department of Health Promotion and Education, Faculty of Public Health, College of Medicine, University of Ibadan, Ibadan, Nigeria

**Keywords:** Knowledge, Perception, Practice, Complementary and alternative medicine, Conventional health care workers, Abuja, Nigeria

## Abstract

**Background:**

Healthcare workers are currently making efforts to offer services that cater to the holistic care needs of their patients. Previous studies have shown that some healthcare workers encounter challenges when advising patients about Complementary and Alternative Medicine (CAM), even though its use is widespread. Many health care workers may not have received formal education or training in CAM and consequently are unable to address their patients’ questions about it. This study explored the knowledge, perception, attitude and practice of CAM by healthcare workers in Garki Hospital, Abuja, Nigeria.

**Methods:**

This was an institution-based cross-sectional study, design and a convergent parallel, mixed methods design was used for data collection. Five (5) healthcare workers were purposively selected as participants for the key informant interviews, while two hundred and fifty (250) selected using a simple random sampling method completed the questionnaire. The data collection instruments used were a key informant interview guide and a 35-item self-administered questionnaire. Knowledge was assessed with a 4-item scale with a maximum score of 8. Perceptions and attitudes were assessed using Likert scales with a maximum score of 45 and 20, respectively. Practice was assessed with a 6-item scale with a maximum score of 18. Qualitative data was analysed using framework analysis. Quantitative data was analysed using descriptive and inferential statistics. Data acquired from both methods were integrated to form the findings.

**Results:**

The average age of respondents for the quantitative study was 34.0 ± 7.8 years, and they were predominantly females (61.2%) with one to ten years of work experience (68.8%). The mean knowledge, perception and attitude scores were 1.94 ± 1.39, 13.08 ± 2.34 and 32.68 ± 6.28, respectively. Multiple linear regression result showed that knowledge (t = 2.025, *p* = 0.044) and attitude (t = 5.961, *p* = 0.000) had statistically significant effects on the practice of CAM. Qualitative data revealed that the majority of the participants perceive CAM favourably, provided it is properly introduced into mainstream medicine with evidence of safety and research to prove its efficacy.

**Conclusion:**

The study has shown the gaps in knowledge and the practices of CAM by conventional medical practitioners. This has implications for their ability to counsel and refer patients who may require CAM therapies. Policy, research and programmatic initiatives that seek to enhance their knowledge of CAM, and improve collaboration with CAM practitioners are recommended.

**Supplementary Information:**

The online version contains supplementary material available at 10.1186/s12906-024-04429-x.

## Background

Complementary and Alternative Medicine (CAM) is “a system of complex medical and health care practices and products that are not generally considered part of conventional medicine” and has been used since antiquity [1]. As centuries passed, the practice receded with more emphasis on the practice of conventional medicine [2]. Recently, CAM has gained increasing attention and interest from healthcare workers and the general public [3]. The use of CAM persists in local communities, especially in low-income countries [1]. Tertiary institutions in some high-income countries have taken steps to integrate CAM curricula into their medical education system [2, 4]. The decision to recommend CAM therapy to a patient is related to the healthcare worker’s knowledge and training [5].

The healthcare system in Nigeria will benefit from increased communication between conventional medicine practitioners and CAM practitioners as both play complementary roles in healthcare delivery, especially given community members’ favourable views on the accessibility and affordability of CAM [6–8]. Legally, the Medical and Dental Practice Act states that CAM practitioners are not authorised to practice medicine and are liable to be punished [9]. However, special provisions (that allow for the supervised, regulated practice of CAM) are made for them under the National Primary Health Care Development Agency (NPHCDA) Act, whose agency was set up to promote CAM’s relevance in the advancement of primary health care [9]. The recorded efforts at integrating CAM into the health care system are selected training of CAM practitioners and public health campaigns with the purpose of training CAM practitioners to adopt good agricultural practices and sound medical practices [9]. These were conducted by the Federal Ministry of Health and international development partners such as the World Health Organisation [10].

Few studies have been conducted on CAM among conventional healthcare workers in Nigeria; all these were quantitative studies [6, 7]. This study explored the knowledge, perception, attitude and practice of CAM by health care workers in Garki Hospital Abuja, Nigeria.

The findings can contribute to the fulfilment of the Sustainable Development Goal (SDG 3) of Good Health and Well-being by creating an enabling environment for patients seeking care for health problems by offering them the choice of using conventional medicine and/ or CAM.

## Methods

### Study design

This was an institution-based, cross-sectional study and a convergent parallel, mixed methods design was used for data collection. The quantitative approach was a cross-sectional study while the qualitative was key informant interviews. Both the quantitative and qualitative data were equally important and occurred concurrently.This approach was adopted to support the collection of complimentary data and enrich the interpretation of the results. The qualitative and quantitative data were analysed separately, and the results were integrated to generate conclusions.

Data collection comprised key informant interviews and a descriptive cross-sectional survey among conventional healthcare workers in Garki Hospital, Abuja, Nigeria. The study assessed healthcare workers’ knowledge, perception, attitude and practice of CAM. The study also identified factors [11, 12] that may influence the healthcare workers’ perception of CAM and its integration into the healthcare system [13].

### Description of the study area

Garki is an urban area located in Abuja, in the Federal Capital Territory of Nigeria, and the main languages spoken are English and Hausa. However, people from many different ethnicities populate the area. The residents engage in various economic activities ranging from banking, health care, civil service, public service, business and telecommunications. The study was limited only to the healthcare workers in Garki Hospital Abuja. Garki Hospital Abuja is a tertiary hospital located in Garki Local Government Area (LGA) of the Federal Capital Territory Abuja Municipal Area Council.

The number of health workers in this institution was 500 as of the time of this study. These consist of 137 doctors (including consultants, senior registrars, registrars and medical officers), 156 nurses, 21 pharmacists, 20 medical lab scientists, six physiotherapists and others including 82 patient care attendants, five preventive medicine counsellors, 10 radiographers, nine medical lab technicians, three optometrists, two embryologists, two psychologists, 10 renal technicians, one dietician and five mortuary attendants.

### Study population

The study population comprised medical doctors (physicians and surgeons), nurses, pharmacists, medical lab scientists, physiotherapists and other healthcare workers working in Garki Hospital Abuja, Nigeria. The inclusion criteria were at least one year of experience post-qualification, complete registration with the appropriate professional bodies and employment as permanent staff. Key informant interviews were also conducted with heads of units of medicine, surgery, nursing, medical laboratory science and pharmacy.

### Sample size determination

The sample size was calculated using the standard formula (derived from Cochran’s formula and Slovin’s formula) [14].

$$n = \frac{{\frac{{{z^2}p\,\left( {1--p} \right)}}{{{e^2}}}}}{{\frac{{1 + ({z^2} * \,p\,\left( {1--p} \right)}}{{{e^2}N}}}}$$

Where:

n = the required sample size.

z = 1.96 (95%) standard normal deviation at the required confidence interval.

p = proportion of health care providers with a favourable attitude toward CAM.

q = 1 – p.

e = margin of error set at 0.05.

*p* = 60.0% (proportion of physicians with a favourable attitude toward CAM in Lagos University Teaching Hospital)^7^.

N = known population of health care workers in Garki Hospital that is 500.

*n* = 213.

A non-response rate of 10% of the minimum sample size was calculated to address possible cases of loss or incomplete completion of the questionnaire.

n_a =_ n x 1/1 – r.

where: n_a_ is the adjusted sample size, n is the initial sample size calculated using standard formula, r is the expected non-response rate (expressed as a decimal).

n_a_ = 213 × 1/1–0.1.

= 213 × 1/0.9.

= 213 × 1.11.

x2009;237

Therefore, the minimum sample size estimate for the study is 237, which was increased to 250.

### Sampling procedure

The healthcare workers were stratified into the different cadres of healthcare. The sample population was selected using a simple random method. The proportionate allocation method was applied in sample selection; the proportion of healthcare workers selected from each subgroup was determined by their number relative to the entire population. The ratio used in the proportionate allocation of health workers into the study is shown in additional file 1.

Five healthcare workers who participated in the key informant interviews were selected through a purposive method. The decision to include them was based on their position as heads of key service delivery units and their level of experience.

### Study instruments

A ten-item key informant interview guide (Additional file 2) was developed from previous studies [2, 15–19] and used to gather information on the knowledge, perception, attitude, practice, challenges, and facilitators to incorporating CAM and the recommendations for the same. The interviewer -administered interviews were conducted to illustrate the perspectives and opinions of experienced authority figures on their perception and attitudes towards CAM.

Quantitative data was collected using a 35-item self-administered questionnaire (Additional file 3) developed from a literature review [2, 5, 7, 17–19]. This instrument included sections covering the socio-demographic characteristics, knowledge of CAM therapies [2, 5, 7], attitude and perception towards CAM therapies [17–19], and practice [2, 19].

### Training of research assistants, pretesting of tools and data collection

Before data collection, the researcher had a two-day orientation with two members of staff of Garki Hospital who had degrees in the social sciences, and they were engaged as research assistants in the study. The orientation focused on the study’s objectives, interview techniques, procedures for data collection and ethical issues. Soon after, the quantitative and qualitative research tools were pre-tested in a nearby hospital with similar characteristics to the study area and revised as appropriate before the conduct of the actual study.

Data collection was conducted between 12th September 2020 and 12th November 2020. For the quantitative data, 250 respondents completed the self-administered questionnaire while interviews were held with five key informants by the researcher.

### Analysis of quantitative and qualitative data

Responses in each questionnaire were coded using a coding guide developed by the research team. This coding guide includes scores for variables to be analysed. The variable, knowledge, was assessed using four questions, and the maximum score for knowledge was 8. For each question, If the respondent answered “Yes” with valid examples, a score of 2 was given; “Yes” without correct examples was assigned a score of 1 and responses that were either “No” or “Don’t know” were given a score of 0. Perception was assessed using four questions on a Likert scale, and the score ranged from 0 to 20. Attitude was also assessed using nine (9) questions on a Likert scale and the score ranged between 0 and 45. The knowledge, attitude and practice were reported using the mean and standard deviation. Practice was assessed using a 6-item scale, and the questions included ever use of CAM by the health worker and counselling and referral of patients for CAM services. The highest and lowest scores were 0 and 18, respectively.

Analysis of quantitative data was done using SPSS version 26. Continuous variables were summarised using mean and standard deviation. Categorical variables such as age, knowledge, perception and practice were grouped into categories derived from the coding and scoring guides. Independent t-test, one-way analysis of variance, pearson correlation and multiple linear regression analysis were used to determine associations and test statistical significance at *p* < 0.05.

For the qualitative data, the audio recordings were transcribed verbatim into Microsoft Word document and analysed using framework analysis to identify common themes supported by normative quotes [20].

This involved a 5-step process: familiarisation with the data, development of a thematic framework, indexing, charting, mapping and interpretation [20]. During the familiarisation process, members of the research team, read the transcripts to get acquainted with the data; after that, there was a discussion of the data. A thematic coding framework was developed based on the discussion during this phase. After that, portions of the transcripts were indexed by identifying the themes and codes where they belong. These were charted by arranging the information in a table according to the themes with the aid of Microsoft Word. Finally, the mapping and interpretation were done by arranging and discussing the charted information on perception, attitude and factors influencing the utilszation of CAM. At this point, the researchers were interested in deducing explanations and patterns across the data.

There was integration and synthesis of the qualitative and quantitative data sets [21]. This helped deepen understanding of CAM among conventional healthcare practitioners providing a more detailed qualitative description. Themes from the quantitative and qualitative data sets were compared to identify areas of differences or commonalities. The data from both sources were integrated during final data interpretation using the weaving approach, which entails a narrative description and presentation of both the qualitative and quantitative findings by themes [21].

## Results

### Participants’ profile (key informant interviews)

For the interviews, the participants’ ages ranged from 38 to 53 years. The results further showed that 60% of the participants were male. 40% were either Idoma or Igbo, and the remaining 20% were Hausa. All participants were Christian and had between two to thirteen years of experience in a supervisory role. The selected individuals include a consultant physician, a consultant surgeon, a senior nurse, a senior pharmacist and a preventive medicine counsellor working under the jurisdiction of the Institute of Human Virology, Nigeria (IHVN).

### Socio-demographic characteristics of respondents

The results showed that the mean age was 34.0 ± 7.8 years and the highest age group proportion (81.2%) was for those aged 20 to 39 years. The results showed that the majority (61.2%) of the respondents were female. Most of the respondents were either medical doctors 41.6% or nurses 29.6%. The data showed that the majority of the respondents, 68.8% had between one to ten years of work experience. The results are shown in Table 1.

**Table 1:** Socio-demographic characteristics of respondents

Variables	Frequency (*N*)	**Percent (%)**
	(*N* = 250)	
**Age (in years)**		
20–29	77	30.8
30–39	126	50.4
≥ 40	47	18.8
**Gender**		
Male	97	38.8
Female	153	61.2
**Religion**		
Christian	214	85.6
Islam	30	12
Traditional African Religion	2	0.8
None	4	1.6
**Profession**		
Medical Doctor	104	41.6
Nurse	74	29.6
Pharmacist	13	5.2
Medical Lab Scientist	9	3.6
Physiotherapist	3	1.2
Paramedical	47	18.8
**Ethnicity**		
Hausa	26	10.4
Igbo	83	33.2
Yoruba	46	18.4
Idoma	13	5.2
Others	82	32.8
**Years of Experience**		
1–10	172	68.8
11–20	59	23.6
21–30	15	6
31–41	4	1.6

Paramedical professions include Optometrists, Embryologists, Radiographers, Dieticians, Renal Technicians and Psychologists

### Knowledge of CAM

The results in Table 2 show the respondents’ knowledge of CAM. The mean knowledge score for the study population is 1.94 ± 1.39 with a range of 0 to 6 from a total possible score of 8 points.

Most (59.6%) had read materials on CAM. Respondents were then asked if they knew the names of alternative/traditional medicines used by practitioners and to list at least three. The majority (65.2%) answered “No” or “Don’t Know”, about one quarter (24.4%) answered “Yes,” and the remaining (10.4%) were able to list correct examples of the medicines that were asked. A high percentage (70.4%) were aware of the risks associated with CAM use.

According to the qualitative findings, most of the participants were unaware of changes within the health system, including government policies favouring the use of CAM or training sessions to instill the knowledge of CAM though they acknowledge a few efforts by governmental and non-governmental organizations to promote integration of CAM as illustrated in these quotes:

“*There have been efforts but everything has to go through a process. It has been delayed. However, there are NGOs and other private bodies that are readily advocating for CAM to be used in the professional health system. But it has not yet been approved.*” Key informant.

*“In the past, there probably were efforts to bring CAM into the mainstream (conventional medicine) but has not seen the light of day due to the problem of effecting policies in Nigeria.”* – Key informant.

**Table 2:** Respondents’ knowledge of CAM

Variable	Frequency	(%)
	(*N* = 250)	
*Read CAM Materials*		
Yes	149	59.6
No	101	40.4
*Know the names of CAM used by practitioners*		
Yes, with correct examples		
Yes	26	10.4
No	61	24.4
	163	65.2
*Aware of CAM therapies listed by NAFDAC*		
Yes, with correct examples		
Yes	15	6
No	16	6.4
	219	87.6
*Aware of risks associated with CAM*		
Yes		
No	176	70.4
	74	29.6

### Perception of complementary and alternative

The results in Table 3 show the respondents’ perceptions of CAM. The mean perception score of the respondents is 13.08 ± 2.34 with a range of 4 to 19, out of a total possible score of 20.0. Less than half, 47.2%, of the respondents, disagree with the statement that healthcare systems should rely on conventional medicine alone. Less than two-thirds (56.2%) of the respondents either agree or strongly agree with the statement that healthcare systems should provide conventional medicine and CAM at the patients’ discretion. A similar percentage (55.2%) agreed or strongly agreed with the statement that healthcare systems should provide conventional medicine and CAM at the healthcare providers’ discretion. The majority of the respondents (73.2%) agree or strongly agree that healthcare systems should provide conventional medicine and evidence-based CAM as integrative medicine.

According to the qualitative findings, the majority of the participants thought that CAM can be appropriately introduced into mainstream medicine provided there is evidence and a lot of research channelled along that path to make sure it is beneficial for patients as shown in this quote: *“If [CAM is] properly introduced into mainstream medicine and there is evidence and a lot of research channelled along that path to make sure CAM is beneficial for patients, I am totally for it.” – Key informant*.

In addition, there were concerns expressed by most of the participants that some CAM practitioners may not understand the biological or pharmacological basis for the efficacy of their products and the National Agency for Food and Drug Administration and Control (NAFDAC) may not approve the use of some CAM products due to this issue as illustrated in this quote:

*“Hardly will NAFDAC give CAM practitioners license to practice. Some practitioners cannot defend [do not know the* pharmacological basis for the efficacy of their products] *what they are giving out. It (CAM practice) will only be done well if it is done the right way.”* – Key informant.

**Table 3:** Perception of the respondents on the future of CAM

Perception Statement	Strongly Agree *N* (%)	Agree *N* (%)	Neither agree nor disagree *N* (%)	Disagree *N* (%)	Strongly disagree *N* (%)	Total *N* (%)
*Healthcare systems should rely on conventional medicine alone*	9 (3.6)	42 (16.8)	52 (20.8)	118 (47.2)	29 (11.6)	250 (100)
*Healthcare systems should provide conventional medicine and CAM at the patient’s discretion*	29 (11.6)	114 (45.6)	47 (18.8)	46 (18.4)	14 (5.6)	250 (100)
*Healthcare systems should provide conventional medicine and CAM at the healthcare provider’s discretion*	21 (8.4)	117 (46.8)	55 (22.0)	45 (18.0)	12 (4.8)	250 (100)
*Healthcare systems should provide conventional medicine and evidence-based CAM as integrative medicine*	45 (18.0)	138 (55.2)	43 (17.2)	19 (7.6)	5 (2.0)	250 (100)

### Attitude towards complementary and alternative

Table 4 shows the respondents’ attitude toward CAM. The mean attitudinal score of the respondents is 32.68 ± 6.28 with a range of 9 to 45, out of a total possible score of 45 points. Almost half (48%) of the respondents strongly agree or agree with the statement that practising with knowledge of CAM and Conventional Medicine is superior to practising with only the knowledge of conventional medicine. The majority (95%) of the respondents agree or strongly agree that research on the efficacy and safety of CAM should be performed. Most, (76.8%) of the respondents agree or strongly agree that medical practitioners should be more educated in the use of CAM.

According to the qualitative findings, some of the potential adverse effects of CAM, which all the participants expressed, include concerns that it will affect conventional medicine practice leading to interferences when a patient decides to explore and use both CAM and conventional medicine causing harm to the patient as illustrated in this quote:

*“There will be interferences because a patient will decide to explore and try both CAM and conventional medicine. This may be harmful to the patient. In terms of our unit (HIV unit managed by the Institute of Human Virology of Nigeria), we try to make our patients understand the repercussions of doing both (CAM and conventional medicine).”* – Key informant.

Another concern expressed by some participants was potential resistance by healthcare providers to accept and provide care using CAM since they were not trained on it. This could hinder acceptability by the health workers, as shown in this quote:

*“We still have a long way to go because the training in conventional medicine has made us believe that if it is not conventional, it shouldn’t be accepted.”* – Key informant.

The positive effects expressed by all the participants include that CAM would be cheaper than conventional medicine, and in a low-income country like Nigeria, it would help people access care at a cost they can afford. Also, it would lead to greater patient acceptability because most of their clients are raised within the traditional Nigerian setting, and a lot still believe in it. In addition, compliance will be enhanced; patient load and satisfaction will increase, as illustrated in these quotes:

*“It will make health care delivery much more affordable, and the health of many Nigerians will be taken care of, meaning cheaper and less resource intensive.”* – Key informant.

">

“*It would lead to greater patients’ acceptability because most of our clients are raised within the traditional Nigerian setting, and a lot still believe in it.”* – Key informant.

### Practice of CAM

The mean practice score of the respondents is 9.1 ± 2.6 with a range of 4 to 18, out of a total possible score of 20 points. It was shown that 82.4% of the respondents had a poor score on CAM utilisation and outcomes (practice) while 17.6% had a good score on the same. The result presented in Table 5 shows that the majority of the respondents (70.0%) had not used or recommended CAM. In comparison, 26.0% of the respondents and 48% were likely to refer their patients to a CAM practitioner if available at the institution. Only 10.4% of the respondents had ever referred their patients to a CAM practitioner while 83.2% had not done so. The majority of the respondents (69.6%) had discussed the possible benefits of CAM therapy with 0–25% of their patients, while 33.2% of the respondents had discussed the possible harmful outcomes of the same with 0–25% of their patients. Almost half (49.6%) of the respondents said that their patients are the ones to initiate discussion of the benefits and risks of CAM therapy. In contrast, the following proportions said it was either themselves (21.2%) or a third party (23.2%).

**Table 4:** Respondents’ attitude toward CAM

Variable	Strongly Agree *N* (%)	Agree *N* (%)	Neither agree nor disagree *N* (%)	Disagree *N* (%)	Strongly disagree *N* (%)	Total *N* (%)
*Practising with knowledge of CAM and Conventional Medicine is superior to practising with only knowledge of conventional medicine*	35 (14.0)	85 (34.0)	55 (22.0)	55 (22.0)	20 (8.0)	250 (100)
*Incorporation of CAM therapies can result in increased patient satisfaction*	22 (8.8)	121 (48.4)	54 (21.6)	42 (16.8)	11 (4.4)	250 (100)
*CAM therapies can assist in fighting illness*	27 (10.8)	121 (48.4)	76 (30.4)	21 (8.4)	5 (2.0)	250 (100)
*Medical Practitioners should be more educated in the use of CAM*	54 (21.6)	138 (55.2)	30 (12.0)	22 (8.8)	6 (2.4)	250 (100)
*I would support the incorporation of CAM in the undergraduate curriculum of my previous course of study*	34 (13.6)	117 (46.8)	55 (22.0)	36 (14.4)	8 (3.2)	250 (100)
*Incorporation of CAM therapies into the health care systems would enhance patient care*	29 (11.6)	110 (44.0)	74 (29.6)	26 (10.4)	11 (4.4)	250 (100)
*I would support CAM being introduced in a drug formulary*	29 (11.6)	119 (47.6)	59 (23.6)	35 (14.0)	8 (3.2)	250 (100)
*Research on the efficacy and safety of CAM should be performed*	129 (51.6)	101 (40.4)	14 (5.6)	5 (2.0)	1 (0.4)	250 (100)
*Provision of wellness centers using CAM and conventional medicine would benefit patients*	37 (14.8)	128 (51.2)	60 (24.0)	21 (8.4)	4 (1.6)	250 (100)

**Table 5:** Practice of CAM

Variable	Frequency	Percent
	(*N*)	(%)
*Used/recommended complementary and*	*Alternative*	
*Medicine*		
Yes	65	26.0
No	175	70.0
Don’t Know	10	4.0
*Likely to refer a patient to a CAM practitioner (if available*		
*at institution) for treatment of an ailment*		
Extremely likely	22	8.8
Somewhat likely	98	39.2
Neither likely nor unlikely	45	18.0
Somewhat unlikely	47	18.8
Extremely unlikely	38	15.2
*Ever referred a patient to a CAM practitioner*		
Yes	26	10.4
No	207	93.2
No Response	17	6.8
*Percentage of patients that health worker has discussed*		
*The possible benefits of using CAM therapies*		
0–25%	174	69.6
26–50%	35	14.0
51–75%	30	12.0
76–100%	11	4.4
*Percentage of patients that health worker has discussed*		
*possible harmful outcomes of using CAM therapies*		
0–25%	83	33.2
26–50%	54	21.6
51–75%	72	28.8
76–100%	41	16.4
*Individual who usually initiates discussion of the benefits and risks of*		
*CAM therapy*		
Self	53	21.2
Patient	124	49.6
Third-party	58	23.2
No response	15	6.0

### Potential barriers and facilitators to the incorporation of CAM into the Hospital practice

A barrier expressed by some of the participants to the incorporation of CAM into Hospital practice includes health care workers not readily aligning with CAM because the institution they work in has not made provisions for it as reflected in these quotes:

*“People [Health workers] will not want to accept it at first because they do not understand it. Garki Hospital has an already established conventional medical practice.”* – Key Informant.

“*Garki Hospital is located in an urban area. The management will not look too kindly on anything that will decrease patient patronage. Practitioners themselves will not readily align with considering CAM.”* – Key informant.

A potential facilitator identified by one of the participants was the availability of financial resources which must be channeled to the conduct of research to prove that these therapies are beneficial and not as harmful.

Another facilitator identified was education. Specifically, a two-pronged approach was emphasised that includes patient education and practitioner education to improve the acceptability of CAM as stated in the quotes below:

*“We need to develop a two-pronged approach consisting of patient education and practitioner ………which will improve acceptability of CAM.”* –Key informant.

*“Mainstream conventional medicine took several years to be accepted so factors such as education, education, education! It is important to educate people on the positive side (of CAM).” –* Key informant.

### Association between the socio-demographic characteristics of respondents and their perception of CAM

Table 6 shows there is no relationship between the respondent’s gender, years of experience, ethnicity, profession and their perception towards CAM. The exception is religion (*P* < 0.05).

**Table 6:** Relationship between the socio-demographic characteristics of the respondents and perception of CAM

Variable *N* = 250	Perception of CAM (Scores)			ANOVA/ T-test (F/T)	*P*
**Age**	**Mean**		**SD**	0.001	0.979
20–29	13.25		2.34		
30–39	13.01		2.34		
≥ 40	13.02		2.34		
**Gender**				1.212	0.272
Male	13.29		2.34		
Female	12.11		2.33		
**Years of Experience**				0.739	0.391
1–10	13.04		2.34		
11–20	13.03		2.34		
≥ 21	12.74	2.33			
**Ethnicity**				0.210	0.647
Hausa	12.38		2.34		
Igbo	13.31		2.33		
Yoruba	13.5		2.34		
*Others	12.87		2.33		
**Religion**				5.707	0.018
Christian	11.55		2.31		
Islam	12.2		2.32		
Traditional African Religion	14	2.34			
None	11.25	2.30			
**Profession**				0.741	0.390
^#^Medical Doctors	12.83		2.32		
Nurses	13.58		2.34		
Pharmacists	14.46	2.35			
**Paramedical Professions	12.61		2.31		

*Other ethnicities include Idoma, Igala, Edo/Ishan, Ebira, Tiv, Ibibio, Efik, Urhobo, Ijaw, Bini, Tarok, Yala, Boki, Akwa Ibom, Angas, Mwaghavul, Clip, Isodas, Nupe, Ebekwara, Ron, Dogmak, Kanuri, Annane, Delta, Oron, Anang, Bassa and Bajju

^#^ Surgeons and physicians

** Paramedical professions include Medical laboratory scientists, Physiotherapists, Optometrists, Embryologists, Radiographers, Dieticians, Renal Technicians and Psychologists

***Significant p (< 0.05)

### Association between the socio-demographic characteristics of respondents and their practice of CAM

There is no relationship between the respondent’s age, gender, ethnicity, years of experience and their practice of CAM. The exceptions are religion and profession (P ≤ 0.05). The result of this finding is shown in Table 7.

**Table 7:** Relationship between the socio-demographic characteristics of the respondents and the practice of CAM

Variable *N* = 250	Practice of CAM (Scores)		ANOVA/ T-test	*P*
**Age**	**Mean**	**SD**	2.410	0.122
20–29	8.58	2.70		
30–39	9.38	2.41		
≥ 40	9.78	2.6		
**Gender**			0.080	0.777
Male	9.15	2.79		
Female	9.05	2.48		
**Years of Experience**			0.762	0.383
1–10	8.98	2.60		
11–20	9.27	2.43		
≥ 21	8.85	3.14		
**Ethnicity**			0.124	0.725
Hausa	9.50	2.97		
Igbo	8.84	2.77		
Yoruba	9.11	2.85		
*Others	8.85	2.38		
**Religion**			0.293	0.034
Christian	9.10	2.54		
Islam	9.17	2.61		
Traditional African Religion	12.00	8.49		
None	7.00	1.15		
**Profession**			0.178	0.027
Medical doctors	8.54	2.38		
Nurse	9.86	2.86		
Pharmacist	9.46	2.47		
**Paramedical Professions	9.54			

*Other ethnicities include Idoma, Igala, Edo/Ishan, Ebira, Tiv, Ibibio, Efik, Urhobo, Ijaw, Bini, Tarok, Yala, Boki, Akwa Ibom, Angas, Mwaghavul, Clip, Isodas, Nupe, Ebekwara, Ron, Dogmak, Kanuri, Annane, Delta, Oron, Anang, Bassa and Bajju

** Paramedical professions include Medical laboratory scientists, Physiotherapists Optometrists, Embryologists, Radiographers, Dieticians, Renal Technicians and Psychologists

***Significant p (< 0.05)

#### Hypothesis 1

There is no significant relationship between the healthcare provider’s knowledge of CAM and their likelihood of incorporating it into their practice.

The results of the findings are shown in Table 8. The table shows that there is a positive correlation between the respondents’ knowledge of complementary and alternative medicine and the likelihood of incorporating it into their practice (*r=.206*, *p* = .001). Therefore, the null hypothesis is rejected.

#### Hypothesis 2

There is no association between the healthcare provider’s attitude toward CAM and their likelihood of incorporating it into their practice.

There is a positive correlation between the attitude of the respondents towards CAM and their likelihood of incorporating it into their practice (*r=.428,* *p* = .000). The result of this finding is shown in Table 8. This means that the attitude of the respondents towards complementary and alternative medicine has a significant influence on their likelihood of incorporating it into their practice. Therefore, the null hypothesis is rejected.

#### Hypothesis 3

There is no significant relationship between the healthcare provider’s perception of CAM and their likelihood of incorporating it into their practice.

The results of the findings are shown in Table 8. The table shows that there is a positive correlation between the respondents’ perception of complementary and alternative medicine and the likelihood of incorporating it into their practice (*r=.215,* *p* = .001). Therefore, the null hypothesis is rejected.

The results showed that the multiple linear regression model was statistically significant (F = 20.067, *P* = 0.000, adjusted R2 = 0.187); knowledge (t = 2.025, *p* = 0.044) and attitude (t = 5.961, *p* = 0.000) had statistically significant effects on the practice of CAM as shown in Table 9.

**Table 8:** Pearson correlation analysis

	Knowledge	Attitude	Perception	Practice
Knowledge	1	0.216 (*P* < 0.005)	0.100 (*P* < 0.005)	**0.206 (** ***P*** ** = 0.001)**
Attitude	0.216 (*P* < 0.005)	1	0.465 (*P* < 0.005)	**0.428 (** ***P*** ** = 0.000)**
Perception	0.100 (*P* < 0.005)	0.465 (*P* < 0.005)	1	**0.215 (** ***P*** ** = 0.001)**
Practice	**0.206 (** ***P*** ** = 0.001)**	**0.428 (** ***P*** ** = 0.000)**	**0.215 (** ***P*** ** = 0.001)**	1

**Table 9:** Multiple linear regression analysis of the factors influencing practice of CAM

Variable	Partial regression coefficient (B)	Standard error (SE)	Standardized partial regression coefficient (beta)	t	*P*
Knowledge	0.222	0.109	0.119	2.025	0.044
Attitude	0.163	0.027	0.392	5.961	0.000
Perception	0.024	0.072	0.021	0.332	0.740
Constant	3.040	0.953		3.191	0.002

## Discussion

Findings from this study revealed that the healthcare workers had poor knowledge about CAM and this had implications on practice. This aligns with finding in the North West [6] and South West [7] regions of Nigeria which showed that the knowledge of CAM is low and related to the healthcare providers’ years of experience. Most had read CAM materials but the majority did not know the products. In addition, the majority were unaware of therapies listed/approved by the National Agency for Food and Drug Administration and Control and only a tenth knew the name of CAM used by practitioners. Several studies among conventional healthcare practitioners in different regions of the world have documented poor knowledge about CAM [5, 22–24]. This finding can be attributed to the fact that many of the respondents may not have been taught CAM during their training nor had they come across such knowledge in the years since graduation. The poor knowledge of CAM has grave implications for the competencies of healthcare workers to counsel, address concerns and provide proper guidance to the increasing number of patients who may be contemplating using CAM therapies or integrative medicine [5]. This is essential because patients form their health beliefs largely on the advice of the health care provider [5]. This finding underscores the need for further education for this cadre of health workers, particularly medical doctors, and this can be implemented through the introduction of CAM courses in medical education institutions [25, 26] or through in-service training [27].

Conversely, the majority of the respondents had a positive perception and attitude towards CAM. Studies in the North West and South West regions of Nigeria have also documented a positive attitude towards CAM among physicians [6, 7]. Respondents in this study expressed that conventional medical practitioners should be more educated on the use of CAM including its possible inclusion in the undergraduate curriculum of their previous course of study. This finding is similar to what was obtained in studies by Hilal et al., and Yurtseven et al. [5, 28].

Majority of the respondents expressed their reluctance to refer their patients to a CAM practitioner due to concerns about the safety and efficacy of the drug. This is similar to findings of a study conducted among physicians in Lagos, Nigeria which revealed that despite a good knowledge of the commonly used herbal preparations, skepticism remained about the value of CAM. Most indicated that they would discourage patients from taking these therapies [7]. Reasons for this were insufficient research conducted on CAM therapies with a lack of data on efficacy and safety of the same. Similar findings were reported in Sokoto [8] where the respondents showed a high degree of concern about the safety of CAM.

The majority of respondents in this study had never used nor referred patients to CAM practitioners, nor had they discussed the benefits of using CAM therapies. These echo the findings from a study conducted in Sokoto, Nigeria, among healthcare workers [6]. This is an expected finding because most healthcare workers have not been trained on CAM and must follow the ethics of their profession and rely on evidence-based research [13] to guide their practice. From this study, some factors affecting the perception of CAM are the respondent’s religion and profession. This is similar to the findings of a study conducted among healthcare workers in Trinidad and Tobago [17].

Potential barriers and challenges to the acceptance of CAM were highlighted. Most respondents believe that healthcare workers are more likely not to accept CAM because they are under the authority and follow the etiquette of sound medical practice in the institution [10]. Another barrier is that patients using the hospital are well-educated; some will not even consider its use. Others cited a lack of standardisation of CAM practice as a reason why it would be difficult to incorporate it into their hospital practice [11]. All these potential barriers and challenges must be considered, especially for patients who may desire to utilise CAM services [12].

The implications of these findings are that there is need for effective integration of CAM into practice that will lead to overcoming communication challenges with patients, addressing potential safety issues and skepticism about CAM efficacy. Addressing these implications requires ongoing education and training to improve healthcare workers’ understanding and approach to CAM, enabling them to provide more comprehensive and patient-centered care.

### Limitations

There are two main limitations to this study. The first is that nurses and healthcare assistants make up the majority of healthcare workers in Garki Hospital and the proportional allocation in the initial sampling reflected this. However, the response rate for these cadres was very low prompting the researchers to administer more questionnaires to the medical doctors to achieve the sample size. The second is that this study was carried out among healthcare workers in one tertiary hospital in Abuja the capital city of Nigeria.

Further studies could consider including healthcare workers in different hospitals to cut across the primary, secondary and tertiary healthcare facilities in Nigeria. This will ensure the results can be generalised.

## Conclusion

This study investigated healthcare workers’ knowledge, perception, attitude and practice towards CAM. The findings have shown the gaps in knowledge and the poor utilisation or referral for CAM services by conventional medical practitioners. Other potential barriers and challenges which may hinder the acceptance of CAM were highlighted. Therefore, the relevant government agencies and professional associations should implement policy, research and programmatic initiatives that enhance CAM and improve collaboration with CAM practitioners.

### Electronic supplementary material

Below is the link to the electronic supplementary material.

Supplementary Material 1

Supplementary Material 2

Supplementary Material 3

## Data Availability

All qualitative and quantitative data generated during and/or analysed during the current study are currently not publicly available but are available from the corresponding author on request. This can only be used for non-commercial purposes which ensures that participants’ confidentiality is protected.

## Abbreviations

CAM: Complementary and Alternative Medicine
FMOH: Federal Ministry of Health
HOD: Head of Department
IHVN: Institute of Human Virology, Nigeria
LGA: Local Government Area
NAFDAC: National Agency for Food and Drug Administration and Control
NANTMP: National Association of Nigeria Traditional Medicine Practitioners
NPHCDA: National Primary Health Care Development Agency
NIPRD: Nigerian Institute of Pharmaceutical Research and Development
SPSS: Statistical Package for the Social Sciences
TCAM: Traditional Complementary and Alternative Medicine
WHO: World Health Organisation
