# Supplementary material for: Knowledge, perception, attitude, and practice of complementary and alternative medicine by health care workers in Garki hospital Abuja, Nigeria
Source: BMC Complement Med Ther. 2024 May 9;24:177. doi: 10.1186/s12906-024-04429-x (PMC11080117; doi:10.1186/s12906-024-04429-x)
Supplement: Supplementary file 1 — Supplementary Material 1 [file 12906_2024_4429_MOESM1_ESM.docx]

**Proportionate sampling of respondents**

| Health care professional | Total Population | Proportionate sample of subgroup selected | Ratio of sample of subgroup selected |
| --- | --- | --- | --- |
| Medical doctors (including physicians and surgeons) | 137 | 74 (104) | 0.540 (0.76) |
| Nursing Officers | 156 | 88 (74) | 0.564 (0.474) |
| Pharmacists | 21 | 13 | 0.619 |
| Medical Lab Scientists | 20 | 9 | 0.45 |
| Physiotherapists | 6 | 3 | 0.5 |
| **Paramedical Professions** |  |  |  |
| Optometrists | 3 | 2 | 0.667 |
| Embryologists | 2 | 1 | 0.5 |
| Radiographers | 10 | 6 | 0.6 |
| Medical Lab Technicians | 9 | 5 | 0.556 |
| Patient Care Attendants | 81 | 52 (28) | 0.642 (0.346) |
| Preventive medicine counselors | 5 | 5 | 1 |
| Dietician | 1 | 1 | 1 |
| Renal Technicians | 10 | 6 | 0.6 |
| Psychologists | 3 | 2 | 0.667 |
| **TOTAL** | 495 (500) * | 267 (259) | 0.534(0.518) |

The sample size was increased to 267 to make room for respondents that would refuse to participate in the survey as well as respondents that would not complete the questionnaire. The figures in brackets represent the number of respondents that actually filled the questionnaire. Then 250 of the completed questionnaires were selected for analysis purposes.

(500) * This is taking into account 5 mortuary attendants that were not included in the study but are also part of the 500 individuals classified as health care professionals in the study area.
