# Supplementary material for: Knowledge, perception, attitude, and practice of complementary and alternative medicine by health care workers in Garki hospital Abuja, Nigeria
Source: BMC Complement Med Ther. 2024 May 9;24:177. doi: 10.1186/s12906-024-04429-x (PMC11080117; doi:10.1186/s12906-024-04429-x)
Supplement: Supplementary file 2 — Supplementary Material 2 [file 12906_2024_4429_MOESM2_ESM.docx]

KEY INFORMANT INTERVIEW GUIDE

**1. Socio-Demographic Background of Respondents**

a)Gender:

b) Religion:

c) Designation:

d) Job Responsibilities at the Hospital

e) Years of service at the Hospital:

2. Tell me about your organisation.

(mission? How long it has been established? Services provided etc)

3. Comment freely on health care delivery in your organisation (types of healthcare services provided; time of operation, client load/volume etc.)

4. Are you aware of any new developments taking place in the health system?

(Government policies that favour the use of complementary and alternative medicine/ programmes run for the purpose of training health care workers on the use of complementary and alternative medicine in healthcare organisations)

5. What is your perception on the introduction of CAM in medical/health care settings?

6. What are the potential positive or negative effects of incorporating complementary and alternative medical (CAM) therapy into Garki Hospital practice (patient satisfaction, client load, health workers’ resistance to new approaches, health worker’s acceptance and satisfaction etc).

7. Discuss the potential challenges/barriers to the incorporation of complementary and alternative medical (CAM) therapy into Garki Hospital practice.

8. Discuss the facilitators to the incorporation of complementary and alternative medical

(CAM) therapy into Garki Hospital practice and other health care settings nationwide.

9. Would you consider the possibility that complementary and alternative medicine practitioners are willing to reveal the source of their medicines?

10. Proffer recommendations for the incorporation of complementary and alternative medical therapy into Garki Hospital practice.

Thank you very much for your time.
