# Supplementary material for: Knowledge, perception, attitude, and practice of complementary and alternative medicine by health care workers in Garki hospital Abuja, Nigeria
Source: BMC Complement Med Ther. 2024 May 9;24:177. doi: 10.1186/s12906-024-04429-x (PMC11080117; doi:10.1186/s12906-024-04429-x)
Supplement: Supplementary file 3 — Supplementary Material 3 [file 12906_2024_4429_MOESM3_ESM.docx]

**QUESTIONNAIRE**

SECTION A. DEMOGRAPHICS OF HEALTH CARE PROFESSIONAL

1. Please state your age in years as at last birthday.

…………………………………………………………………………………………

2. Please state your gender

[ ] Male [ ] Female

3. Please state the number of years you have practiced

…………………………………………………………………………………………

4. Please state your ethnicity

[ ] Hausa [ ] Igbo [ ] Yoruba [ ] Other, please specify

…………………………………

5. Please state your religious affiliation

[ ] Christianity [ ] Islam [ ] Traditional African Religion [ ] None [ ] Other, please

specify…………………………………………………………………………………

6. State your profession

[ ] Physician [ ] Surgeon [ ] Nurse [ ] Pharmacist [ ] Medical Lab Scientist [ ] Physiotherapist [ ] Other, please specify…………………………………………………………………….

7. Time dedicated to patient care in percentage (%) [ ] 0 – 25 [ ] 26 – 50 [ ] 51 – 80 [ ] 81 – 100

SECTION B. KNOWLEDGE AND PRACTICE OF COMPLEMENTARY AND ALTERNATIVE MEDICINE (CAM)

1. I have read materials on complementary and alternative medicine

[ ] Yes [ ] No [ ] Don’t know

2. I know the names of some alternative/ traditional medicines used by practitioners

[ ] Yes [ ] No [ ] Don’t know

Please list at least

three…………………………………………………………………………

3. I am aware of some of the alternative / traditional medicines listed for use by NAFDAC

| [ ] Yes | [ ] No | [ ] Don’t know |  | |
| --- | --- | --- | --- | --- |
| Please |  | list | at | least |

three………………………………………………………………………….

4. I am aware of any risks associated with the use of complementary/ alternative/

traditional medicine

[ ] Yes [ ] No [ ] Don’t know

SECTION C. ATTITUDES TOWARDS COMPLEMENTARY AND ALTERNATIVE MEDICINE (CAM)

1. Practicing with knowledge of CAM and Conventional Medicine is superior to practicing with only knowledge of conventional medicine

[ ] Strongly agree [ ] Agree [ ] Neither agree nor disagree [ ] Disagree [ ] Strongly disagree

2. Incorporation of CAM therapies can result in increased patient satisfaction

[ ] Strongly agree [ ] Agree [ ] Neither agree nor disagree [ ] Disagree [ ] Strongly disagree

3. CAM therapies can assist in fighting illness

[ ] Strongly agree [ ] Agree [ ] Neither agree nor disagree [ ] Disagree [ ] Strongly disagree

4. Medical Practitioners should be more educated in the use of CAM

[ ] Strongly agree [ ] Agree [ ] Neither agree nor disagree [ ] Disagree [ ] Strongly disagree

5. I would support incorporation of CAM in the undergraduate curriculum of my previous course of study

[ ] Strongly agree [ ] Agree [ ] Neither agree nor disagree [ ] Disagree [ ] Strongly disagree

6. Incorporation of CAM therapies into the health care systems would enhance patient care

[ ] Strongly agree [ ] Agree [ ] Neither agree nor disagree [ ] Disagree [ ] Strongly disagree

7. I would support CAM being introduced in a drug formulary

[ ] Strongly agree [ ] Agree [ ] Neither agree nor disagree [ ] Disagree [ ] Strongly disagree

8. Research on the efficacy and safety of CAM should be performed

[ ] Strongly agree [ ] Agree [ ] Neither agree nor disagree [ ] Disagree [ ] Strongly disagree

9. Provision of wellness centers using CAM and conventional medicine would benefit patients

[ ] Strongly agree [ ] Agree [ ] Neither agree nor disagree [ ] Disagree [ ] Strongly disagree

SECTION D. PERCEPTION OF THE FUTURE OF CAM

1. Health care systems should rely on conventional medicine alone

[ ] Strongly agree [ ] Agree [ ] Neither agree nor disagree [ ] Disagree [ ] Strongly disagree

2. Health care systems should provide conventional medicine and CAM at the patients’

discretion

[ ] Strongly agree [ ] Agree [ ] Neither agree nor disagree [ ] Disagree [ ] Strongly disagree

3. Health care systems should provide conventional medicine and CAM at the health care providers discretion

[ ] Strongly agree [ ] Agree [ ] Neither agree nor disagree [ ] Disagree [ ] Strongly disagree

4. Health care systems should provide conventional medicine and evidence - based CAM

as integrative medicine

[ ] Strongly agree [ ] Agree [ ] Neither agree nor disagree [ ] Disagree [ ] Strongly disagree

SECTION E. CAM UTILISATION AND OUTCOMES

1. I have recommended complementary and alternative medicine to a patient. [ ] Yes [ ] No [ ] Don’t know

2. How likely are you to refer a patient to a CAM practitioner (if available at your institution) for treatment of an ailment?

[ ] Extremely likely [ ] Somewhat likely [ ] Neither likely nor unlikely

[ ] Somewhat unlikely [ ] Extremely unlikely

3. Have you ever referred a patient to a CAM practitioner? [ ] YES [ ] NO [ ] No Response

4. With approximately what percentage of your patients do you talk about possible

benefits of using CAM therapies?

[ ] 0 – 25 [ ] 26 – 50 [ ] 51 – 75 [ ] 76 – 100

5. With approximately what percentage of your patients do you talk about the possible harmful outcomes of using CAM therapies?

[ ] 0 – 25 [ ] 26 – 50 [ ] 51 – 75 [ ] 76 – 100

6. Who usually initiates discussion of benefits and risks of CAM therapy? [ ] Self [ ] Patient [ ] Third party

SECTION F. ACCEPTANCE OF CAM

1. I have initiated a discussion about CAM with a colleague?

[ ] Never [ ] Rarely [ ] Sometimes [ ] Often [ ] Very often

2. I feel comfortable discussing CAM with patients.

[ ] Never [ ] Rarely [ ] Sometimes [ ] Often [ ] Very often

3. I have initiated a discussion about the interaction between CAM and conventional medicine with a colleague.

[ ] Never [ ] Rarely [ ] Sometimes [ ] Often [ ] Very often

4. I would discourage patients from taking CAM.

[ ] Strongly agree [ ] Agree [ ] Neither agree nor disagree [ ] Disagree [ ] Strongly disagree

5. Complementary and alternative medicine practitioners should be given the chance to practice.

[ ] Strongly agree [ ] Agree [ ] Neither agree nor disagree [ ] Disagree [ ] Strongly disagree
